# Structure Design and Performance Study of Bionic Electronic Nasal Cavity

**DOI:** 10.3390/biomimetics10080555

**Published:** 2025-08-21

**Authors:** Pu Chen, Zhipeng Yin, Shun Xu, Pengyu Wang, Lianjun Yang, You Lv

**Affiliations:** 1 College of Engineering and Technology, Jilin Agricultural University, Changchun 130118, China; 20240338@mails.jlau.edu.cn (P.C.); 20240293@mails.jlau.edu.cn (Z.Y.); baibq1822@mails.jlu.edu.cn (P.W.); zhenyuw24@mails.jlu.edu.cn (L.Y.); 2 Changchun Satellite Observation Station, National Astronomical Observatories, Chinese Academy of Sciences, Changchun 130117, China; lvyou@cho.ac.cn

**Keywords:** electronic nose, bionic chamber, sensors, soil pesticide residue

## Abstract

A miniaturised bionic electronic nose system was developed to solve the problems of expensive equipment and long response time for soil pesticide residue detection. The structure of the bionic electronic nasal cavity is designed based on the spatial structure and olfactory principle of the sturgeon nasal cavity. Through experimental study, the structure of the nasal cavity of the sturgeon was extracted and analyzed. The 3D model of the bionic electronic nasal cavity was constructed and verified by Computational Fluid Dynamics (CFD) simulation. The results show that the gas flow distribution in the bionic chamber is more uniform than that in the ordinary chamber. The airflow velocity near the sensor in the bionic chamber is lower than in the ordinary chamber. The eddy current intensity near the bionic chamber sensor is 2.29 times that of the ordinary chamber, further increasing the contact intensity between odor molecules and the sensor surface and shortening the response time. The 10-fold cross-validation method of K-Nearest Neighbor (K-NN), Random Forest (RF) and Support Vector Machine (SVM) was used to compare the recognition performance of the bionic electronic nasal cavity with that of the ordinary electronic nasal cavity. The results showed that, when the bionic electronic nose detection system identified the concentration of pesticide residues in soil, the recognition rate of the above three recognition algorithms reached 97.3%, significantly higher than that of the comparison chamber. The bionic chamber electronic nose system can improve the detection performance of electronic noses and has a good application prospect in soil pesticide residue detection.

## 1. Introduction

In the process of agricultural production, pesticide is very important for the prevention and control of pests and diseases and the improvement of food yield [1]. At the same time, however, the overuse of pesticides also causes heavy pesticide residues in soil. Liquid chromatography is commonly used for soil pesticide residue detection [2,3]. But the interference of the test sample from sampling to detection cannot be effectively removed. Liquid chromatography instruments face difficulties in tackling the issues of temporal efficiency and quantitative detection [3]. For volatile pesticide residues, analytical methods such as gas chromatography-mass spectrometry (GC-MS) and mass spectrometry (MS) are widely used [4]. Among them, GC-MS combines the separation capability of gas chromatography with the qualitative accuracy of mass spectrometry, providing high sensitivity and specificity for volatile compounds; mass spectrometry, as a powerful tool for molecular weight determination and structural characterisation, can achieve accurate quantification of trace volatile residues. However, these methods usually require complex sample pre-treatment, long analysis times and rely on specialised operations, resulting in their limited applicability in rapid on-site testing scenarios. International and Chinese national standards [5] set stringent maximum permitted concentrations (MPCs) for pesticide residues in soil: the limits for organophosphorus pesticides (e.g., chlorpyrifos) in agricultural soils are usually 0.01–0.1 mg/kg, and for carbamate pesticides (e.g., carbaryl) 0.05 mg/kg. These strict limits highlight the need for accurate and sensitive detection, even minor exceedances can cause ecological or food safety problems, which adds to the complexity of the analysis [6]. Within the field of fluid and gas detection technologies, electronic nose systems have garnered substantial interest in both research and application domains [7]. An electronic nose system is a detection technology that uses a gas sensor array to detect sample gas for analysis and identification. In recent years, it has been widely used in food science, medicine, agricultural science, environmental monitoring and other fields. The electronic nose has the characteristics of fast detection speed and wide measurement and evaluation range, which can meet the requirements of timeliness and accuracy of soil pesticide residue detection to a certain extent [8,9,10].

Meanwhile, a range of accomplishments in the sampling and separation of soil pesticide residues have laid a foundation for the detection of such residues via electronic noses [11]. Nevertheless, owing to the extensive planting areas and intricate environmental conditions, detection equipment must possess high sensitivity and strong adaptability. With the development of the electronic nose, bionics interacts with the electronic nose and gradually forms a frontier discipline. The olfactory perception system of organisms inspires the electronic nose system. The functionality of biological olfactory sensing is contingent upon several factors, including nasal cavity structure, the quantity of olfactory cells, and the diffusion of odor molecules in air and mucous membranes. Consequently, altering the internal structure or volume of the nasal cavity can markedly enhance olfactory performance [12]. Thus, the design of electronic nose structures that mimic the nasal cavity’s structure and function exerts a significant influence on the miniaturization, sensitivity, and stability of electronic nose systems. In recent years, many scholars have designed different electronic nose systems by imitating the nasal structure of different organisms to improve the performance of electronic nose systems. Professor Lorena Villarreal [13] proposed a ventilated artificial nostril design for odor perception by studying the human nostril structure. The device improves the response characteristics of the sensor by simulating human nostril inhalation, sampling and exhalation. Wang Jiayin’s team [14] designed a handheld electronic nose bionic chamber for liquor recognition by studying the structural characteristics of the mammalian nasal cavity. Professor Chang Zhiyong designed a new electronic nasal cavity structure by studying the human turbinate structure and combining shark skin’s V-shaped groove structure [15]. By applying the structure of the dog’s nasal cavity to the structure design of the electronic nasal cavity, Weng Xiaohui optimised the design of a small electronic nasal cavity suitable for underground oil and gas detection and improved the performance of the electronic nose system [16]. In addition, the team of Mostafa Shooshtari and Alireza Salehi developed an electronic nose based on a CNT-TiO_2_ hybrid structure for detecting VOCs. CNTs were prepared by the PECVD method, and TiO_2_ nanowires were grown by the hydrothermal method, which increased the sensitivity by three times and shortened the response time by 30 s. A virtual array was constructed with four types of electrode, and combined with temperature modulation and other techniques; the recognition accuracy for four types of VOCs reached 97.5% [17].

The flow of shallow water carries odorants that trigger the sturgeon’s sense of smell, and its nasal structure has evolved to exhibit excellent shunting and diffusing capabilities [18]. The nasal cavity of sturgeon is more suitable for soil pesticide residue detection than the olfactory system of other organisms [19]. The nasal cavity of mammals (e.g., dogs, humans) has a complex turbinate structure with increased volume that hinders miniaturisation [20]. The olfactory system of insects, although sensitive to specific volatiles, lacks versatility for the detection of multiple pesticides. Other fish (e.g., carp) can adapt to odourants dissolved in water but are less efficient at capturing airborne volatiles. In contrast, sturgeon, as an anadromous migratory fish in shallow waters, often interacting with the air–water interface, have a nasal structure that is adaptable to both dissolved odour substances and volatile gases. Their multibranched channels have variable cross sections that enhance turbulence for uniform odour diffusion, which is critical for capturing trace pesticides in soil. In addition, their small size allows for quick interaction with sensors to meet just-in-time requirements. The nasal cavity structure of sturgeons exhibits excellent adaptability for electronic nose system design. In this study, a novel bionic electronic nasal cavity structure was developed by imitating the sturgeon’s nasal architecture. Specifically, considering the environmental characteristics and detection requirements of soil pesticide residues, a structure optimization design method was adopted for chamber engineering. By establishing a fluid–structure interaction control model, the effectiveness and detection accuracy of the bionic electronic nose were experimentally verified. This study offers both theoretical backing and empirical groundwork for the subsequent use of electronic nose systems in detecting soil pesticide residues.

## 2. Materials and Methods

### 2.1. Bionic Nasal Cavity Design

#### 2.1.1. Bionic Nasal Cavity External Structure Design

**Figure 1:**
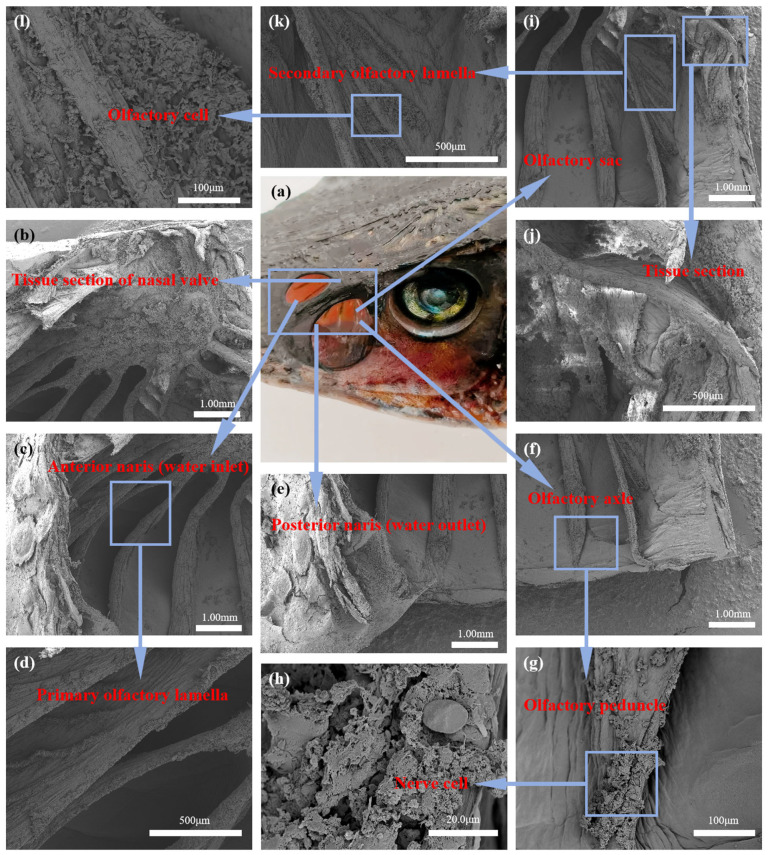
SEM Micrograph of the Sturgeons’ Nasal Cavity, (**a**) is a panoramic view of the sturgeon’s nasal cavity, including (**b**) a cross-sectional view of the nasal valve tissue in the sturgeon’s nasal cavity, (**c**) a view of the anterior nares (water inlet) of the sturgeon’s nasal cavity, (**e**) a view of the posterior nares (water outlet) of the sturgeon’s nasal cavity, (**f**) a diagram of the olfactory ache of the sturgeon’s nasal cavity, and (**i**) a diagram of the olfactory sac of the sturgeon’s nasal cavity. Among these, (**c**) the anterior nares (water inlet) of the sturgeon’s nasal cavity are used to introduce water flow for the perception of chemical signals, and include (**d**) a diagram of the primary olfactory plate of the sturgeon’s nasal cavity; (**e**) the posterior nares (water outlet) of the sturgeon’s nasal cavity are used for efficient drainage to maintain the dynamic balance of the olfactory system; (**f**) the olfactory ache of the sturgeon’s nasal cavity is the core region responsible for efficiently capturing and processing chemical information in water with-in the sturgeon’s olfactory system, including (**g**) the olfactory peduncle of the sturgeon’s nasal cavity and (**h**) the neural cells of the sturgeon’s nasal cavity; (**i**) the olfactory sac in the sturgeon’s nasal cavity is used for efficient capture and conversion of chemical signals in water, including (**k**) the secondary olfactory plate diagram of the sturgeon’s nasal cavity, (**l**) the olfactory cell diagram of the sturgeon’s nasal cavity, and (**j**) the olfactory sac tissue section diagram of the sturgeon’s nasal cavity.

This paper uses scanning electron microscopy (SEM) imaging technology (Hitachi Regulus8100, Tokyo, Japan) to observe the internal structure of sturgeon. The operational process strictly follows the guidance on SEM methodology in the relevant literature [21], healthy adult sturgeons with body lengths of 35–40 cm were selected, and 1 mm^3^ nasal tissue block was taken under aseptic environment, fixed with 2.5% glutaraldehyde fixative (SPI Company, No. 02607-BA for CA, USA) at 4 °C for 12–24 h, rinsed with 0.1 M phosphate buffer (pH 7.0) for 3 times (each time for 15 min), and then rinsed with 1% O_S_O_4_ solution (Ted Pella Inc., No. 012103 for CA, USA) for 1–2 h, followed by 30%, 50%, 70%, 70%, and 30% of O_S_O_4_ (pH 7.4) for 3 times (each time for 15 min). After fixation for 1–2 h with 1% osmium solution (Ted Pella Inc., No. 012103), the samples were rinsed with 0.1 M phosphate buffer (pH 7.4) for 3 times (15 min each time), and then treated with 30%, 50%, 70%, 80%, 90%, 95% ethanol (Sinopharm Corp., No. 100092183 for Shanghai, China) and 100% ethanol for 20 min, and then dried with a Quorom k850 critical point dryer. The samples were dried with a Quorom k850 critical point dryer, fixed on an aluminium sample stage with a conductive carbon adhesive, and then sprayed with platinum for 120 s on a Hitachi MC1000 ion sputtering apparatus to enhance the conductivity, and then finally observed and captured images under a Hitachi Regulus8100, and the results of microstructural observations are shown in Figure 1. Based on these findings, a bionic chamber was designed to mimic the sturgeon’s internal architecture, The bionic chamber design adopts the structure of a “small mouth and large cavity” [22]. The chamber adopts the circular section design, which facilitates the symmetrical arrangement of sensors, and the bionic electronic nose can adapt to more complex environments [23]. Taking into account the dilution effect exerted by an excessively large chamber on gases [24], the minimum section diameter of 12 mm was adopted on the premise that the bionic electronic nose could be connected to the gas storage tank and valve. Secondly, in view of the downsizing of bionic electronic noses and the arrangement of internal sensors [25], the minimum length of the bionic electronic nasal chamber is 110 mm. An orthogonal test determines the size of the bionic cavity of the electronic nose. Four factors (A), cavity length (B), sampling gas flow rate (C) and sampling time (D) of the bionic cavity were selected. Four factors and three horizontal L9(34) orthogonal test factors were established, as shown in Table 1.

**Figure 2:**
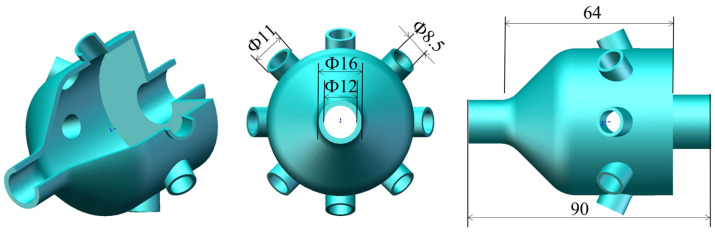
Schematic diagram of the exterior part of the chamber.

Table 1 A2B2C2D1, specifically: the radius ratio of the bionic chamber outer shell’s top section to its minimum section is 4, the electronic nose chamber length is 90 mm, the sampling gas flow rate is 0.8 m·s^−1^, and the sampling time is 60 s. The designed electronic nasal cavity structure is illustrated in Figure 2.

#### 2.1.2. Bionic Nasal Cavity Internal Structure Design

**Figure 3:**
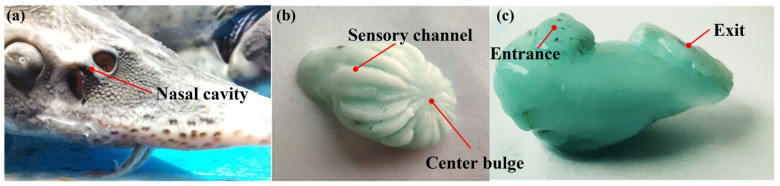
Chamber inverted mold model. (**a**) Panoramic view of the sturgeon’s head, showing the sturgeon’s nasal cavity. (**b**,**c**) Cast models of the sturgeon’s nasal cavity chambers, Sensory channel, Center bulge, Entrance, and Exit.

**Figure 4:**
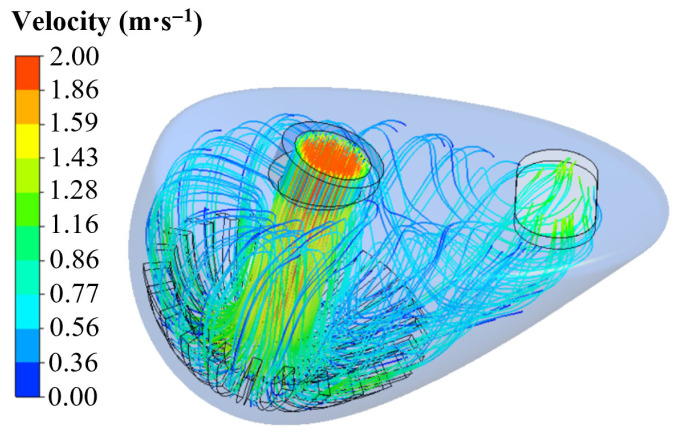
Visualization of water flow direction of nasal structure of sturgeon.

Figure 3 shows the internal model of the Siberian sturgeon in the nasal cavity of the Siberian sturgeon [26]. Then, through a 3D scanner, the internal space digital model of the nasal cavity was established [27], and the fluid kinematics simulation was carried through the nasal cavity by using the model to conduct the Siberian paddlefish [28]. Figure 4 is the chamber flow to the display.

Through flow velocity observations, it was found that water flow reaches the mid-nasal cavity region, where the buffer zone reduces turbulent dispersion of odorants. Finally, under the guidance of the curved nasal wall, the water accumulates and drains out of the nasal cavity [29,30]. This flow structure prolongs the contact time between water flow and odorants in the nasal cavity. Olfactory receptors are embedded within the nasal cavity due to the protective mechanism of the nasal epithelium, which facilitates the transport of chemical substances from the olfactory epithelial region to the olfactory organ via fluid dynamics [31]. As shown in Figure 4, after the water enters the nasal cavity at high speed, it hits the middle of the nasal cavity and generates turbulence. It is dispersed to the olfactory organs of the nasal cavity. The design of the installation and bionic structure of the device is supported by the design of the installation and bionic structure of the bionic electronic nose sensor [16].

The internal structure of the bionic cavity was designed based on the internal structure of the sturgeon nasal cavity and the electronic nose system. Therefore, the center of the electronic nasal cavity is set as a convex structure, and the partitions and grooves around the convex are also set according to the sensory channels in the nasal cavity of the sturgeon to form sensory channels [32]. The dimensions of the four elements, including the diameter of the convex, the number of sensory channels, the depth of grooves and the height of the partition, need to be considered in the interior structure of this cavity.

**Figure 5:**
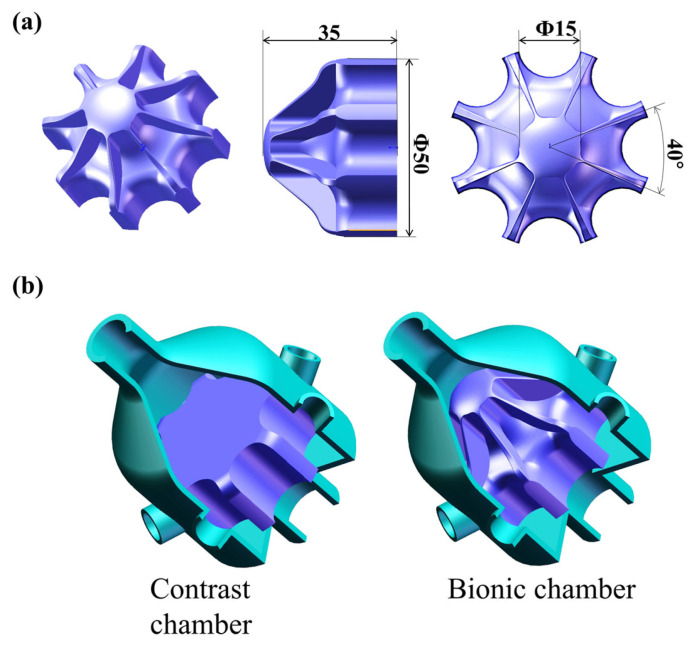
(**a**) bionic structure design of the chamber interior, (**b**) comparison of chamber structure.

Orthogonal tests were used to determine the main dimensions of the boss, partition and groove and the number of sensory channels. Four factors were investigated: boss diameter E, partition height F, groove depth G, and number of sensory channels H. Table 2 serves as the table of factor levels for the L9(34) orthogonal experiment involving six factors with three levels each. The maximum response value detected via electronic noses was adopted as an indicator for performing ANOVA and factor significance testing on the measured data. The parameter settings are the same as the orthogonal test results. Through comparative analysis of different detection conditions, the optimal detection parameter combination was determined as E2F1G2H1, specifically: boss diameter of 15 mm, diaphragm height of 20 mm, groove depth of 15 mm, and 8 sensory channels. The structure of the bionic electronic nasal cavity chamber is depicted in Figure 5a.

In order to verify that the bionic mechanism of the bionic electronic nasal chamber is better than that of the ordinary chamber, a comparison chamber was made in this paper and compared with the bionic chamber. In order to ensure the representativeness of the experimental results, the ratio of the maximum to the minimum sectional radius of the comparison chamber, the length of the chamber, the sensor placement hole and the wall thickness of the comparison chamber were consistent with those of the bionic chamber. In contrast, the structure of the chamber was a platform to remove the bionic parameters of the sturgeon nasal cavity, and the thickness of the platform was consistent with that of the bionic chamber, both being 16 mm. The specific structure is shown in Figure 5b.

### 2.2. Aerodynamic Simulation of Nasal Structure

The model was imported into the Design Modeler (DM) module in ANSYS 2022 R2 software to establish the chamber fluid region, and the meshing module was used for grid division [33]. Considering the structural characteristics of the bionic chamber and common chamber models, a mapping grid was used for grid division [34]. The convex and sensory channels within the chamber were subjected to encryption and refinement. The bionic nasal cavity model yielded 590,718 nodes and 310,985 elements, while the control chamber model had 219,835 nodes and 1,183,854 elements.

The mesh model was imported into the Fluent module, and the Coupled algorithm was selected for calculation [35]. The solver selects the standard K-ε model [36], the operating mode is set as transient, the gas as an incompressible fluid, the initial flow at the inlet is set as 0.8 m/s [37], the free flow boundary condition at the outlet, the outer wall boundary grid layer is set as 3, and the growth rate is set as 1.2. Ten points were extracted from each sensor surface to compare the vortex intensity of the sensor surface in the two chambers.

### 2.3. Bionic Electronic Nose Performance Test

**Figure 6:**
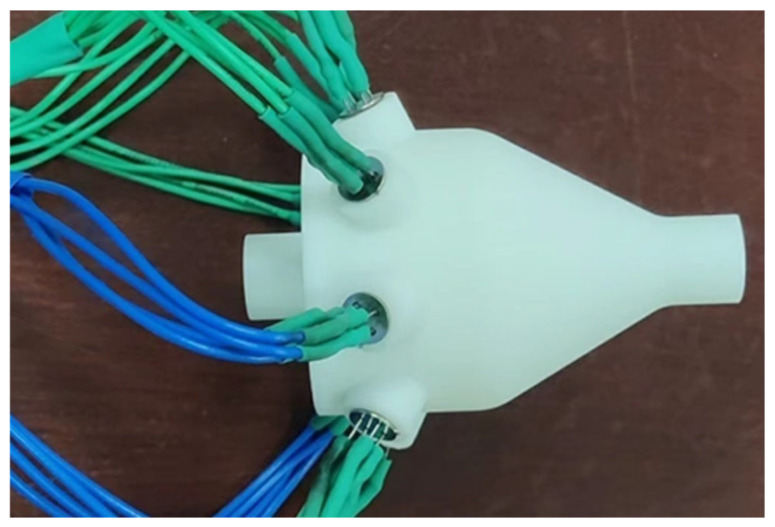
3D printed bionic nasal cavity.

This section describes the preparation of test samples, selection of the electronic detection system, and experimental scheme. The chamber model was fabricated using a Lite600HD 3D printer (Shanghai, China) based on the designed electronic nasal cavity structure. The printing material was 8200 resin, with printing parameters as follows: resolution of 750 × 750 dpi, dimensional accuracy of CT7-CT8, surface roughness Ra of 32 μm. The printing quality of each chamber component met the experimental requirements, as demonstrated by the finished test sample shown in Figure 6. The wires are securely connected to the 3D-printed bionic chamber using heat-shrink tubing.

The experimental soil was taken from the experimental field of Jilin Agricultural University, and herbicides (acetochlor-atrazine [38], glyphosate [39]) and insecticides (chlorpyrifos [40], imidacloprid [41]) used in widely cultivated crops (corn, soybean and rice) in Northeast China were selected as the research objects. Preparation of contaminated soil samples: Select 30 soil samples, add 15 mL of water to each sample, and seal them with food-grade plastic wrap to prevent water evaporation, so as to maintain the relative humidity of the samples at 80 ± 1%. Use a pipette to titrate 1 mL of the corresponding pesticide solution into each sample according to the proportion concentrations shown in Table 3, and stir thoroughly with a glass rod to ensure uniform mixing. Additionally, the prepared pesticide solution should be sealed separately with plastic wrap to prevent odor volatilization from affecting the accuracy of the experimental results. The optimal amount of each pesticide was determined by referring to the pesticide residue standard, and three soil samples with different concentrations were allocated for each pesticide. The concentration distribution of each component is shown in Table 3.

**Figure 7:**
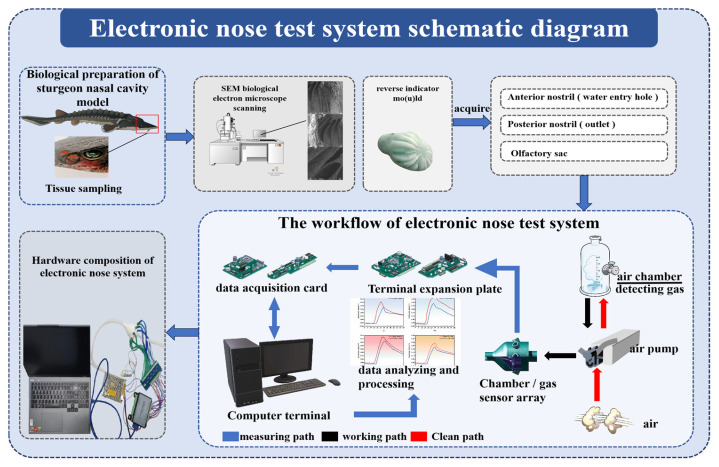
Schematic of electronic nose system: workflow and hardware composition.

Gaseous or volatile testing is accomplished by a bionic electronic nose system. The selection of sensors is based on the characteristics of the gaseous constituents that may be released by the pesticide under test, as well as on the principles of sensor array design (broad-spectrum response, selectivity for specific gases, differentiated and overlapping response characteristics, fast response time, good repeatability). Therefore, sensors TGS2603, TGS2610, TGS2611 and TGS2612, which are more sensitive to gas composition in TGS series gas sensors produced by Figaro Company of Japan, were tested. The target gases measured by the gas sensors and the detection ranges are shown in Table 4. Other hardware parts include a data acquisition card, air pump, expansion board, air chamber, filter and noise reduction circuit, data acquisition instrument, and computer. The data acquisition system is the ART-USB3106A acquisition system of Altai Company, the ART-USB3106A module is a multifunctional data acquisition card manufactured by Altai Company of China, operating at a voltage of 12 V. This board provides 16 RSE/NRSE channels or 8 channels of differential analogue input; 2 channels of analogue synchronous output; 16 programmable I/O channels; and 1 counter channel. The data acquisition card has a total of 40 pins, namely AI0~AI15, P0.0~P1.7, AGND, DGND, and AISE. Among these, P1.4~P1.7 are also designated as PFI0~PFI3. The entire electronic nose testing system is divided into three parts. The first part is the sample introduction module, primarily composed of a sample container and a miniature air pump, responsible for introducing the gases emitted by the tested soil samples and cleaning the chamber; the second part is the detection module, composed of a sampling chamber, sensor array, etc., which is the most critical component of the entire electronic nose testing system, responsible for collecting the chemical signals contained in the detected gases and converting them into electrical signals; the third component is the control module, which includes a data acquisition card, transformer, test circuit, and computer. Its primary function is to transmit and record the sample gas information collected by the detection module after converting the chemical signals into electrical signals. The schematic diagram of the electronic nose system, including its workflow and hardware composition, is shown in Figure 7.

## 3. Results and Discussion

### 3.1. CFD Analysis Results

**Figure 8:**
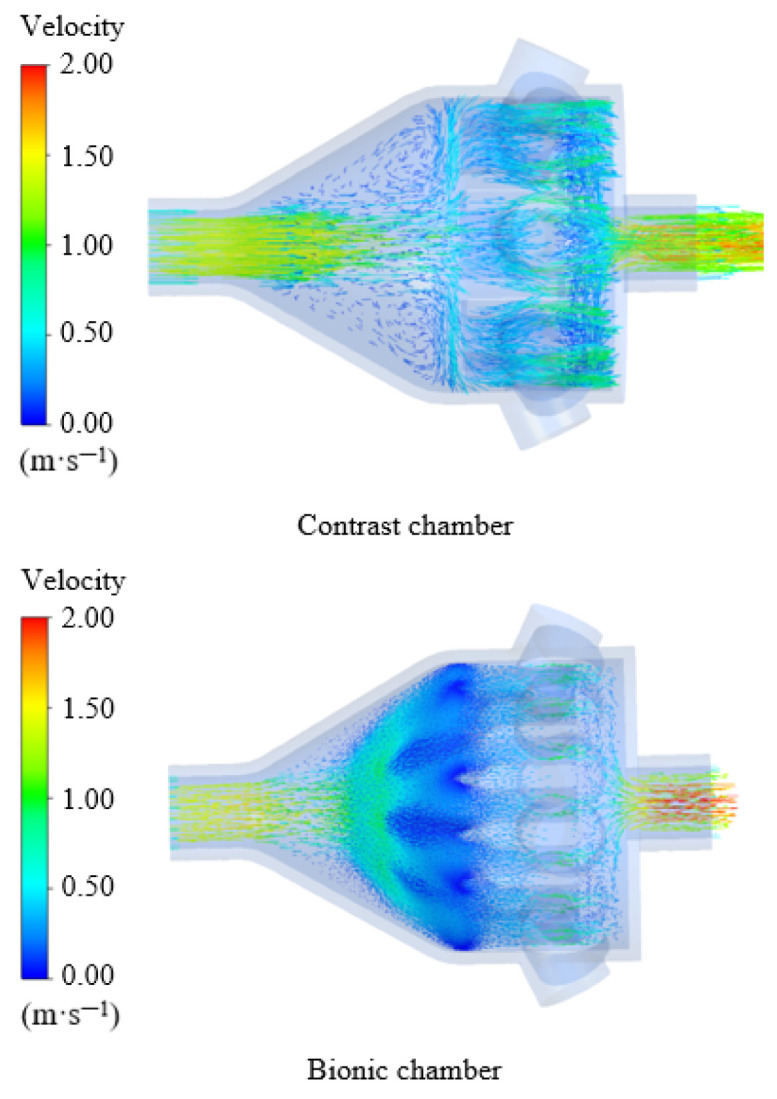
Velocity vector diagram.

The hydrodynamic analysis mainly explores the influence of a bionic chamber on gas flow. After the calculation, CFD-POST is used to post-process the data. Figure 8 shows the velocity vector diagram of the bionic chamber and the ordinary chamber.

**Figure 9:**
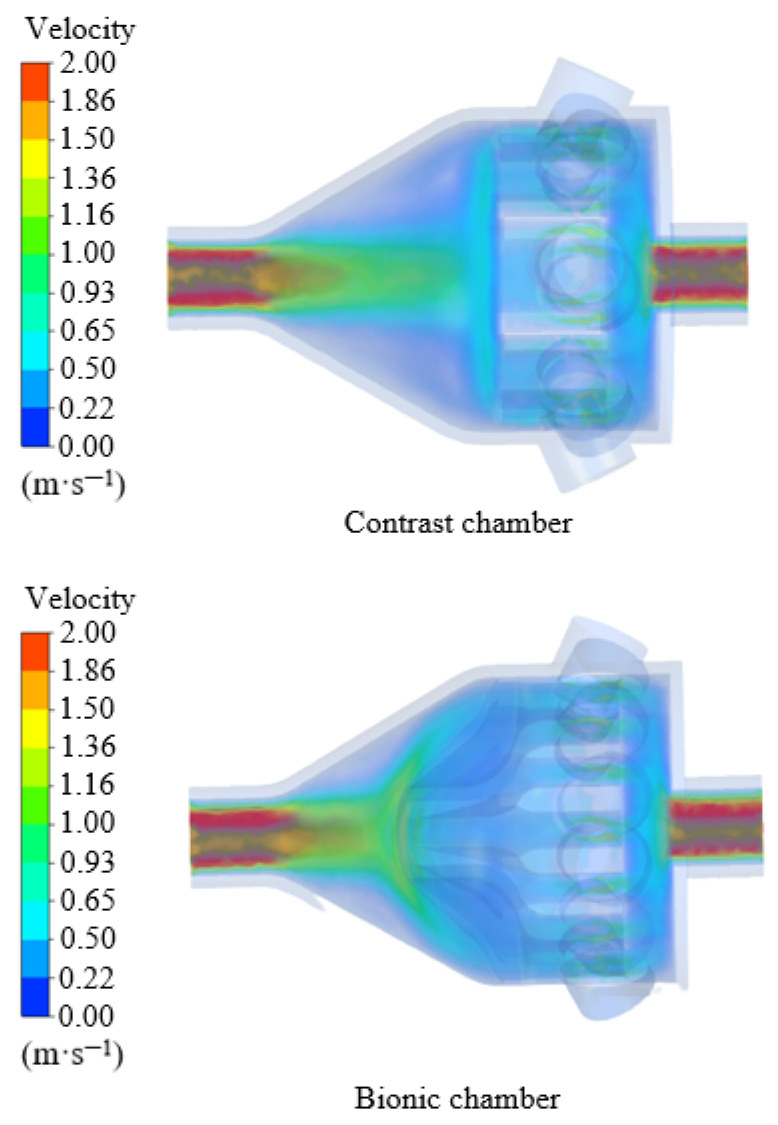
Velocity distribution.

As can be seen from the Figure 9, due to the intervention of the bionic structure, the bionic electronic nasal cavity can quickly diverge the airflow after entering, and the measured gas can completely enter the sensory channel so that the sensor array in the cavity can fully contact the airflow. Moreover, the bionic chamber generates a small gas vortex near the sensory channel, which can increase the gas pressure in the sensory channel and make the gas more fully in contact with the sensor. The simulation results are consistent with those of Agbesi [42]. In the ordinary chamber, the airflow cannot be divided quickly after entering, and only a small part of the measured gas enters the sensory channel. Compared with the recognition results, it can be found that for the bionic chamber, due to the addition of the bionic structure of the sturgeon nasal cavity, the incoming airflow can be quickly shunted, and the time of gas reaching the sensor surface is reduced. It is confirmed that the bionic electronic nasal cavity can receive the gas electronic signal faster and reduce the detection time of the electronic nose system.

**Figure 10:**
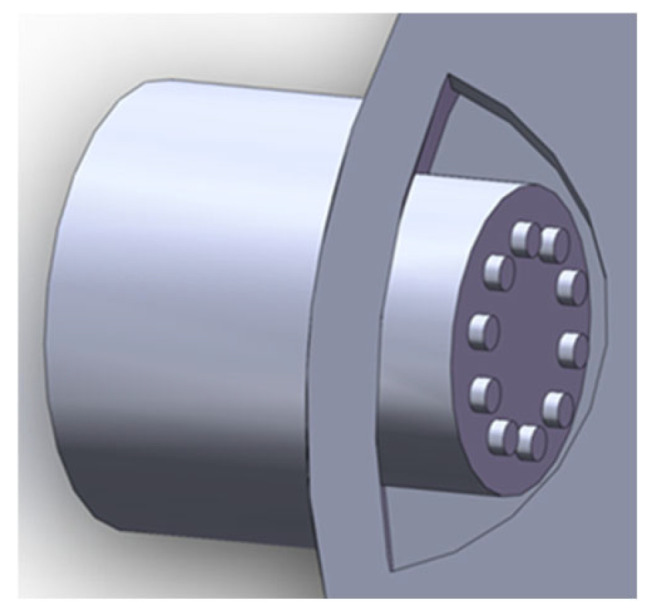
Schematic diagram of position of each point.

In order to better analyze the gas flow in the two-chamber chamber, the velocity distribution cloud image of the two-chamber chamber is obtained by simulation [43]. As can be seen from Figure 10, the airflow velocity in the bionic chamber gradually slows down from the entrance, and the lowest velocity is near the olfactory sensor. In contrast, the airflow velocity in the ordinary chamber is faster. It can be seen from the simulation results that the air velocity on the surface of the olfactory sensor in the bionic chamber is significantly lower than that in the ordinary chamber. Therefore, compared with the ordinary chamber, the measured gas in the bionic chamber is more fully in contact with the sensor in the sensory channel, and the signal intensity of the sensor is higher [19].

Professor Zhao Kai and Professor Pamela Dalton found in the study of human and mouse nasal smell that there are small vortices in the olfactory region of the nasal cavity, which can make odor molecules more fully contact the olfactory cells [31]. Similarly, Russell Garwood found that shallow water entering the nasal cavity of sturgeon also creates a small vortex in the sensory channel so that the olfactory cells in the channel are in full contact with the smell molecules in the water. Suppose a small vortex is created around the sensor in the electronic nasal cavity [26]. Under such circumstances, the sensitivity of electronic noses will likewise be enhanced, while gas molecules can achieve full contact with the sensor surface. In the present study, 10 points on the sensor surface of both the bionic chamber and the ordinary chamber were chosen to compare vortex intensity.

**Figure 11:**
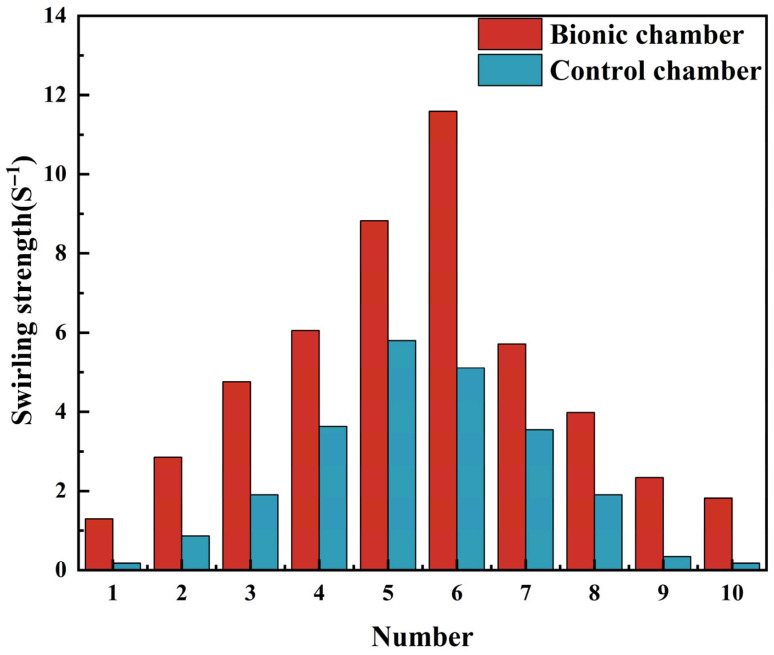
Comparison of vortex intensity.

Figure 10 presents a 10-point positional diagram for vortex intensity analysis, comparing the surface vortex intensity of sensors between the two chambers. As illustrated in Figure 11, the vortex intensity in the vicinity of the sensor within the bionic chamber is markedly greater than that in the ordinary chamber. Simulation findings indicate that the average vortex intensity near the bionic chamber stands at 4.896, whereas the corresponding value for the ordinary chamber is 2.133, demonstrating that the bionic chamber has a 2.29-fold higher vortex intensity in the sensor’s vicinity. This enhancement in vortex intensity facilitates more complete contact between odor molecules and the sensor surface, further validating that the bionic chamber outperforms the control chamber in detection accuracy.

### 3.2. Performance Test Result

#### 3.2.1. Sensor Response Comparison

**Figure 12:**
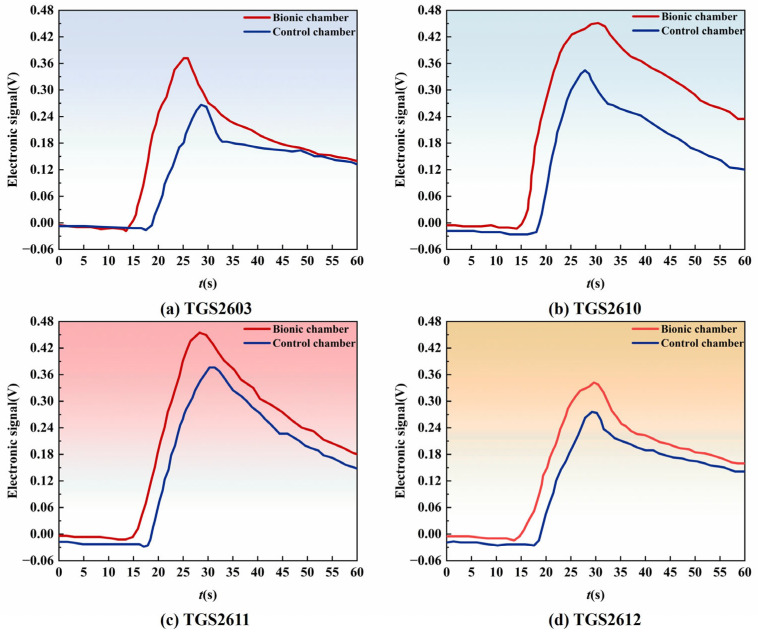
Response curves of four gas sensors in different chambers. The analyte tested was soil samples containing four pesticides (acetochlor-atrazine, glyphosate, imidacloprid, chlorpyrifos) with different concentrations. Each subgraph corresponds to a specific sensor: (**a**) TGS2603, (**b**) TGS2610, (**c**) TGS2611, (**d**) TGS2612. Curves in each subgraph compare the response intensity of the sensor in the bionic chamber (blue) and the control chamber (red) over time (t/s).

In the electronic nose detection of soil pesticide residues, the sensor’s time and degree of response after gas enters the chamber greatly impact the sensitivity of the electronic nose system. When the sensor’s response time is short for an electronic nose detection system, the odor molecular data collected is insufficient, affecting the later data analysis and processing. When the sensor exhibits weak response signals and low sensitivity, the data change will be insignificant, leading to cumbersome post-processing and reduced system recognition rate. To further confirm how two distinct chambers affect the sensor array of electronic noses, the results of data analysis underwent normalization. Additionally, response curves were plotted for the four sensors that exhibited the most significant responses in both chambers, as illustrated in Figure 12. The results show that the response intensity of TGS2603, TGS2610, TGS2611 and TGS2612 installed in the bionic chamber is greater than that of the comparison chamber. After analysis, it is judged that the pressure and speed of the sensor near the olfactory channel are relatively high due to the convex structure in the bionic chamber, and turbulence is formed on the sensor surface. The duration that gas lingers around the sensor is prolonged, and the contact becomes adequate; additionally, the response time and intensity of the gas sensor are enhanced to some degree. This finding demonstrates that the detection sensitivity of the bionic chamber exceeds that of the conventional comparison chamber [44].

#### 3.2.2. Algorithm Recognition Rate Comparison

K-NN (k-Nearest Neighbor), RF (Random Forest) and SVM (Support Vector Machine) learning algorithms established the soil pesticide residue recognition model [45]. The detailed parameters of the machine learning algorithms are set as follows: KNN, number of neighbors K = 5 (to balance overfitting and underfitting), with Euclidean distance adopted as the distance metric; RF: Number of estimators = 100 (balancing performance and computational cost); maximum depth is unrestricted (automatically determined by data); number of split features = square root of the total number of features; SVM: Kernel function = radial basis function; penalty coefficient = 1.0 (balancing misclassification cost and model complexity); kernel coefficient = 1/number of features (calculated automatically based on feature count). Through comparing the performance of the two identification models, the gas chamber and algorithm that are better suited to gas detection and identification under the experimental conditions were chosen. The 780 groups of experimental data were processed by the 10-fold cross-validation method, and ten times 10-fold cross-validation was conducted to obtain the average value. The recognition rate of the two chambers was classified by the following two methods, as shown in Table 5.

It can be found from the calculation results that the detection and recognition rate of the bionic electronic nose system for soil pesticide residues can all reach 97.3% by using the above three learning algorithms, the feature extraction methods listed in Table 6 are defined as follows: IV (Integral Value) denotes the integral value of the sensor response curve, which reflects the cumulative response strength over time; MAX (Maximum) denotes the maximum response value, which represents the peak signal amplitude; Mean denotes the average response value, which characterises the average signal level during the stable response period; WT (Wavelet Transform) denotes the wavelet transform-based feature extraction, which captures the frequency domain features of the signals by using the low-frequency coefficients obtained through the db4 wavelet decomposition. frequency domain features of the signal. These features together provide the classification algorithm with multi-dimensional features of the sensor response, and the specific identification results are shown in Table 6 and Table 7.

By comparing the recognition effect of the three models, it is found that the recognition rate of the electronic nose system installed with a bionic chamber is higher than that of the electronic nose system installed with an ordinary chamber. The influence of the nasal cavity chamber design on the classification rate arises from its indirect enhancement of feature distinguishability through improving sensor response sensitivity. As noted in Section 3.2.1, the bionic chamber optimises gas flow (e.g., generating vortices and extending gas residence time) to significantly improve the response intensity and stability (sensitivity) of the sensors, rather than altering their selectivity (i.e., the inherent preference of sensors for specific gases). This enhanced sensitivity amplifies the differences in sensor response curves between different pesticides or concentrations (e.g., the higher response intensity of TGS2603, TGS2610, TGS2611, and TGS2612 in the bionic chamber), providing more distinguishable feature inputs for classification algorithms (KNN, RF, SVM). Consequently, the algorithms can more accurately identify different sample categories, ultimately resulting in a higher classification rate for the bionic chamber. With respect to the k-nearest neighbor model, the recognition accuracy of the bionic chamber exceeds that of the ordinary chamber by 3.45%. In the case of the Random Forest model, the bionic chamber’s recognition accuracy is 3.25% higher than that of the ordinary chamber. For the Support Vector Machine, the recognition accuracy of the bionic chamber surpasses that of the conventional chamber by 3.0%. Detailed analysis findings are presented in Table 8 and Table 9.

## 4. Conclusions

This article Inspired by the nasal cavity characteristics of Acipenser baeri Brandt, a bionic electronic nasal cavity was designed, and relevant fluid dynamics analysis was conducted to validate its design efficacy. Results demonstrate that the bionic electronic nasal cavity exhibits superior structural performance compared to conventional counterparts. For identical sensor arrays and pattern recognition methods, at the optimal inlet velocity, the bionic system’s sensitivity is significantly enhanced. Specifically, when using the RBF algorithm, the bionic nasal cavity achieves a 3% higher recognition rate than conventional cavities, while the SVM algorithm yields a 3.5% improvement. Fluid analysis confirms that the bionic structure minimises gas residence time in non-sensing regions, reduces flow velocity on the sensor surface, and increases turbulence intensity. Experimental results further indicate that the bionic design positively modulates airflow disturbance within the chamber, thereby enhancing sensor sensitivity and the overall detection performance of the bionic electronic nose system.

Prospect: This study focuses on a biomimetic electronic nose system based on the structure of sturgeon nasal cavity, aiming at the direct detection of volatile pesticide residues (e.g., acetamiprid, atrazine, glyphosate, etc.) in the soil, and does not involve the application of pyrolysis technology. At the technical level, sensor calibration was based on standard samples of known concentration of the target pesticides to ensure detection accuracy by establishing a correspondence between the response signal and the concentration, and was not calibrated against standards for pyrolysed soil organic matter (pyrolyzed SOM). Signal processing uses normalisation to eliminate differences in sensor baseline and response intensity, combined with wavelet transform to extract time-frequency domain features and filter noise to improve signal discrimination. In terms of performance and application, the bionic structure shortens the response time by enhancing the airflow characteristics, and the system is miniaturised (minimum cross-section of 12 mm, length of 110 mm) and 3D printed for portability without complex pre-processing, with an accuracy of 97.3% in pesticide residue detection in the field, which is feasible for large-scale soil testing. In the future, we will expand the combination of pyrolysis technology and electronic nose, explore the pyrolysis temperature range of 300–800 °C and the heating rate of 10–20 °C/min (combined with thermogravimetric analysis optimisation), and establish a standard calibration system for pyrolysis of soil organic matter; conduct in-depth research on the effects of moisture and inorganic compounds on the pyrolysis process, and evaluate the long-term stability of the sensor in the environment of pyrolysis gases; and expand the scope of detection to the detection and determination of Soil Organic Matter (SOM) and its detection of SOM. (SOM) and determine its limit of detection (LOD) and limit of quantification (LOQ), improve the signal processing technology, promote the application of the system in large-scale field monitoring and soil quality census, and form a more comprehensive soil testing technology system.

## Figures and Tables

**Table 1:** Level table of factors for the design of the outer part of the chamber.

Level	Factors			
	A/mm	B/mm	C/(m∙s^−1^)	D/s
1	3.6	80	0.5	60
2	4.0	90	0.8	90
3	4.4	100	1.2	120
Sum of deviation square	0.189	0.059	0.111	0.002
Mean square	0.119	0.056	0.079	0.024
F test value	5.133	1.195	3.171	0.024

**Table 2:** Horizontal table of interior design factors.

Level	Figures			
	E/mm	F/mm	G/mm	H
1	10	20	10	8
2	15	22	15	12
3	20	24	20	16
Sum of deviation square	0.193	0.057	0.132	0.001
Mean square	0.124	0.063	0.086	0.021
F test value	5.253	1.251	2.986	0.021

**Table 3:** Concentration ratio of pesticide components.

Reagent Name	Recommended Usage	Proportioning Concentration		Concentration Value (mg/L)
29% acetochlor 26% atrazine	Dilute 100–150 times	Low	Dilute 100 times	2900 (acetochlor) + 2600 (atrazine)
		Medium	Dilute 40 times	7250 (acetochlor) + 6500 (atrazine)
		High	Dilute 20 times	14,500 (acetochlor) + 13,000 (atrazine)
30%glyphosate	Dilute 95–285 times	Low	Dilute 100 times	3000
		Medium	Dilute 40 times	7500
		High	Dilute 20 times	15,000
45% chlorpyrifos	Dilute 283–353 times	Low	Dilute 300 times	1500
		Medium	Dilute 120 times	3750
		High	Dilute 60 times	7500
5% imidacloprid	Dilute 375–500 times	Low	Dilute 350 times	143
		Medium	Dilute 140 times	357
		High	Dilute 70 times	714

Note: concentration values are based on the active ingredient content of commercial insecticides. For example, “29 per cent acetamiprid” means that the commercial formulation contains 290 g/L of active ingredient. The same calculation is used for other pesticides.

**Table 4:** Target gas measured by gas sensor and detection range.

Sensor Serial Number	Sensor Type	Target Gas	Detection Range (ppm)
1	TGS2603	Organic solvents, organic gases (methane, methyl mercaptan, etc.)	1–30
2	TGS2610	Ethanol, Hydrogen, Methane, Isobutane, Propane	500–10,000
3	TGS2611	Ethanol, Hydrogen, Isobutane, Methane, Natural Gas	500–10,000
4	TGS2612	Methane, Propane, Isobutane	500–10,000

**Table 5:** Classification methods.

Classification Method	Explain
Divided into 4 kinds	Divided into four pesticides (acetochlor-atrazine, glyphosate, imidacloprid, chlorpyrifos)
Divided into 13 kinds	Divided into 13 samples (pesticide type + concentration), such as [acetochlor-atrazine − low concentration], [imidacloprid − medium concentration]

**Table 6:** Chamber recognition rate—divided into four types.

Classifier	Feature Extraction Method	Training Set Recognition Rate		Test Set Recognition Rate	
		Bionic Chamber	Contrast Chamber	Bionic Chamber	Contrast Chamber
KNN	IV	99.7%	100%	99.2%	98.2%
	MAX	100%	100%	99.2%	99.2%
	Mean	100%	100%	99.4%	97.9%
	WT	98.2%	89.4%	97.9%	87.6%
RF	IV	100%	100%	98.9%	97.6%
	MAX	100%	100%	99.2%	96.4%
	Mean	100%	100%	99.4%	98.2%
	WT	100%	93.0%	97.4%	83.0%
SVM	IV	100%	100%	100%	99.4%
	MAX	99.7%	99.6%	99.7%	98.7%
	Mean	100%	100%	100%	99.7%
	WT	99.7%	92.5%	98.9%	88.4%

**Table 7:** Chamber recognition rate—divided into 13 types.

Classifier	Feature Extraction Method	Training Set Recognition Rate		Test Set Recognition Rate	
		Bionic Chamber	Contrast Chamber	Bionic Chamber	Contrast Chamber
KNN	IV	100%	99.8%	97.9%	94.8%
	MAX	100%	99.7%	93.8%	94.6%
	Mean	100%	99.5%	97.6%	94.6%
	WT	100%	86.9%	93.0%	78.9%
RF	IV	100%	99.7%	95.6%	95.1%
	MAX	100%	99.7%	95.3%	95.1%
	Mean	100%	99.7%	95.3%	94.6%
	WT	100%	92.8%	92.5%	77.9%
SVM	IV	99.7%	98.8%	98.2%	96.6%
	MAX	97.2%	99.3%	96.6%	93.8%
	Mean	99.7%	98.8%	98.2%	97.1%
	WT	97.7%	90.2%	95.1%	81.2%

**Table 8:** Divided into four types.

Classifier	KNN		RF		SVM	
Chamber Type	Bionic Chamber	Contrast Chamber	Bionic Chamber	Contrast Chamber	Bionic Chamber	Contrast Chamber
Recognition rate	99.2%	96.5%	99.4%	96.0%	99.75%	97.3%

**Table 9:** Divided into 13 types.

classifier	KNN		RF		SVM	
Chamber Type	Bionic Chamber	Contrast Chamber	Bionic Chamber	Contrast Chamber	Bionic Chamber	Contrast Chamber
Recognition rate	97.8%	93.6%	97.3%	94.3%	98.0%	94.5%

## Data Availability

The original contributions presented in this study are included in the article, and further inquiries can be directed to the corresponding author.
